# Patient Acceptance of Telemedicine in Urogynecology Consultations – A Cross-Sectional Study Performed at a Brazilian Public Institution

**DOI:** 10.1055/s-0042-1748971

**Published:** 2022-06-27

**Authors:** Débora Vianna D'Almeida Lucas Macharet, Leonardo Nogueira Mendes, Walter Carlos Santos de Oliveira, Gláucia Miranda Varella Pereira, Marilene Vale de Castro Monteiro

**Affiliations:** 1 Postgraduate Program in Women's Health of Federal University of Minas Gerais, Belo Horizonte, MG, Brazil; 2 Division of Urology, Hospital das Clínicas of Federal University of Minas Gerais, Belo Horizonte, MG, Brazil; 3 Division of Urology, Hospital das Clínicas of Federal University of Minas Gerais, Belo Horizonte, MG, Brazil; 4 Physiotherapist, State University of Campinas, Campinas, SP, Brazil; 5 Department of Gynecology and Obstetrics, Federal University of Minas Gerais, Belo Horizonte, MG, Brazil

**Keywords:** telemedicina, estudos de viabilidade, preferência do paciente, internet, ginecologia, telemedicine, feasibility studies, patient preference, internet, gynecology

## Abstract

**Objective** To evaluate the acceptance of telemedicine and determine its associated factors in an urogynecology outpatient clinic of a public hospital in Brazil.

**Methods** The present was a cross-sectional study performed between June and November 2020. The included patients had their elective appointments postponed due to the coronavirus disease 2019 (COVID-19) pandemic. The variables considered regarding the acceptance of telemedicine were: urogynecologic diagnosis, age, level of schooling, place of residence, access to the internet, type of device used, frequency of internet use, and use of social media platforms. The categorical variables were described by their absolute and relative frequencies. The association among variables was evaluated through the Fisher exact test, and univariate and multivariate analyses, considering the acceptance of telemedicine as the dependent variable.

**Results** A total of 225 patients were listed, and 182 agreed to participate. The mean age was 59 years old, 81.3% of the patients had access to the internet, and 87.3% of them accepted telemedicine. There were statistically significant associations regarding the acceptance of telemedicine and high levels of schooling ( *p* < 0.01), internet access ( *p* < 0.01), daily use of the internet ( *p* < 0.01), access through personal mobile phone ( *p* < 0.01), and access through the participant's own residence ( *p* < 0.01). In the univariate and multivariate analyses, only high levels of schooling were associated with the acceptance of telemedicine (Adjusted odds ratio: 4.82; 95% confidence interval = 1.59–14.65).

**Conclusion** Most of the urogynecology patients of a public hospital in a developing country accepted telemedicine. Internet access and level of schooling were the factors associated with the acceptance of telemedicine in urogynecology.

## Resumo

**Objetivo** Avaliar aceitação da telemedicina e determinar seus fatores associados em uma clínica de uroginecologia de um hospital público brasileiro.

**Métodos** Trata-se de estudo transversal realizado entre junho e novembro de 2020. Foram recrutadas pacientes que tiveram seus atendimentos eletivos adiados devido à pandemia de doença do coronavírus 2019 ( *coronavirus disease 2019* , COVID-19, em inglês). As variáveis consideradas para a aceitação da telemedicina foram: diagnóstico uroginecológico, idade, escolaridade, local de residência, acesso à internet, tipo de dispositivo, frequência do uso da internet, e uso de plataformas de redes sociais. As variáveis categóricas foras descritas na forma de suas frequências absoluta e relativa. A associação entre essas variáveis foi avaliada por meio do teste exato de Fisher e análises uni e multivariada, e a aceitação da telemedicina foi considerada a variável dependente.

**Resultados** Um total de 225 pacientes foram listadas, e 182 concordaram em participar. A idade média das participantes foi de 59 anos; 81,3% delas tinham acesso à internet, e 87,3% aceitaram a telemedicina. Observaram-se associações estatisticamente significativas entre a aceitação e maior escolaridade ( *p* < 0,01), acesso à internet ( *p* < 0,01), uso diário da internet ( *p* < 0,01), acesso por celular próprio ( *p* < 0,01), e acesso da própria residência ( *p* < 0,01). Nas análises uni e multivariada, somente alto nível de escolaridade esteve associado à aceitação da telemedicina (razão de probabilidades ajustada: 4,82; intervalo de confiança de 95% = 1,59–14,65).

**Conclusão** A maioria das pacientes atendidas em um hospital público de um país em desenvolvimento concordaram com a telemedicina. Acesso à internet e nível de escolaridade foram fatores associados à aceitação da telemedicina nessa população.

## Introduction

Telemedicine is defined as the use of technology to connect a patient to a healthcare provider. 1 The adoption of this form of care, already widely discussed, became even more relevant after the outbreak of the coronavirus disease 2019 (COVID-19) pandemic in early 2020. The need for social distancing brought up the fact that a significant portion of medical consultations can be performed remotely. 2

In this context, there has been an effort to implement and accelerate the use of telemedicine in many fields, with further discussions regarding laws and regulations, and the development of evidence-based protocols to guide remote care.

Regarding urogynecology, a guide 3 was recently published to help clinicians provide high-quality care through remote access. Telemedicine is an opportunity to minimize exposure without sacrificing treatments, and it has opened a new door in the field of urogynecology, mainly concerning the follow-up of treatments even after the pandemic. 4 5 A recent study 6 evaluating telemedicine in the postoperative care of pelvic floor dysfunctions showed that telephone follow-up was not inferior to in-person clinic visits regarding patient satisfaction, and there was no difference in clinical outcomes or adverse events among the groups. The leading urogynecologic diagnoses that may demand follow-up during the pandemic are urinary tract infection, urinary incontinence, and pelvic organ prolapse. 7

In Brazil, before March 2020, telemedicine was only allowed to be used between health care professionals to discuss clinical cases and to promote continuing education in the context of the public health system. The interaction between health professionals and patients was not authorized until an emergency regulation valid only during the COVID-19 pandemic was enacted. 8 9 The health care system in the country is divided between private and public subsystems. Even though the public health system is free and universal, the access to secondary care, such as specialists, is difficult, and often favors those with the ability to pay for the services. 10 Since the regulation, many hospitals have shown interest in implementing telemedicine, but there is no data evaluating how or if public hospitals and their patients are able to use this healthcare modality. There are limiting factors such as the quality of internet connection, the availability of proper devices, and the level of technology literacy among this population. Brazil has more than 212 million inhabitants and has significant levels of social and economic inequality. 11 Among the population aged between 16 and 64 years, there are 140 million social media users, and 94% of them have smartphones. 12 However, women with urogynecological diseases are often older than 60 years, which may be a limiting factor for acceptance and adherence to telemedicine.

Although telemedicine has great potential, there is a lack of data demonstrating its use in Brazil, and we believe it is still underused, especially in the public health system. It is essential to know the resources available, the patterns of internet use, and the interest of the patients in telemedicine to evaluate the feasibility of the implementation of this form of care and how to best approach this population. Furthermore, we intend to demonstrate that patients of the Brazilian public health institutions and those of older ages can engage in this form of care. Therefore, the present study investigates the acceptance of urogynecology telemedicine in a public hospital in Brazil and analyzes its associated factors.

## Methods

The present work was a cross-sectional study performed in a urogynecologic outpatient clinic of a public university hospital in the city of Belo Horizonte, state of Minas Gerais, Southeastern Brazil, from June to November 2020. The study was approved by the institutional review board (under CAAE 41733021.7.00005149), and all participants provided informed consent by telephone.

The inclusion criteria were all patients whose urogynecology medical appointment had been postponed due to the COVID-19 pandemic, who were able to be contacted by telephone call, and agreed to participate. The contact data was extracted from the Hospital's records. The patients who did not agree to participate or could not be contacted were not included in the analysis. The sociodemographic data of those who did not accept telemedicine was analyzed once they agreed to participate in the research. Due to the hospital's strategic plan to combat the COVID-19 pandemic, all urogynecologic appointments were considered elective and canceled from March to November 2020. A sample size of 163 was calculated considering a confidence intercal (CI) of 95%, an α error of 0.05, and an estimated proportion of 88% of agreement with telemedicine, based on previous research 13 evaluating the acceptance of telemedicine in urogynecology.

The data was collected through a review of the medical records and structured interviews conducted by telephone. We tried to reach the patients three times, at different times of the day and different days of the week. Two members of the staff of the outpatient clinic performed the interviews, the data was recorded using the Redcap (Vanderbilt University, Nashville, TN, United States) software, and the telephone calls were not recorded nor transcribed. The variables considered regarding telemedicine acceptance were selected based on a previous systematic review conducted by Scott Kruse et al. 14 (2018): primary urogynecologic diagnosis, age, level of schooling, place of residence, access to the internet, type of internet connection, place of access, type of device used, frequency of internet use, and use of social media platforms. The level of schooling was considered “;low”; if the patients did not have any formal education or had not concluded the equivalent of middle school in the brazilian education system; and “;high”; if they had concluded middle school or had higher degree of education (high school or college degree). The questions related to internet use and social media platforms could have more than one single answer.

The patients were asked if they agreed with telemedicine care for their condition. We defined this care as a remote appointment made by telephone or video call instead of an in-person visit. They were divided into two groups: those who accepted telemedicine (group 1) and those who did not (group 2).

The statistical analysis was performed using the Statistical Package for the Social Sciences (IBM SPSS Statistics for Window, IBM Corp., Armonk, NY, United States) software, version 21.0. The categorical variables were expressed as absolute and relative frequencies, and age, by the mean and median values. Sociodemographic, clinical, and internet-use variables were tested using the Fisher exact test, and univariate and multivariate logistic regression tests were used to identify possible associations with the agreement or disagreement with telemedicine care. Two continuous variables were converted to binary variables following clinically-relevant criteria: age was categorized as < 50 years or > 51 years, based on the increased prevalence of urogynecological symptoms after this age; and the level of schooling was divided into low or high. Values of *p* < 0.05 were considered statistically significant.

## Results

**Fig. 1:**
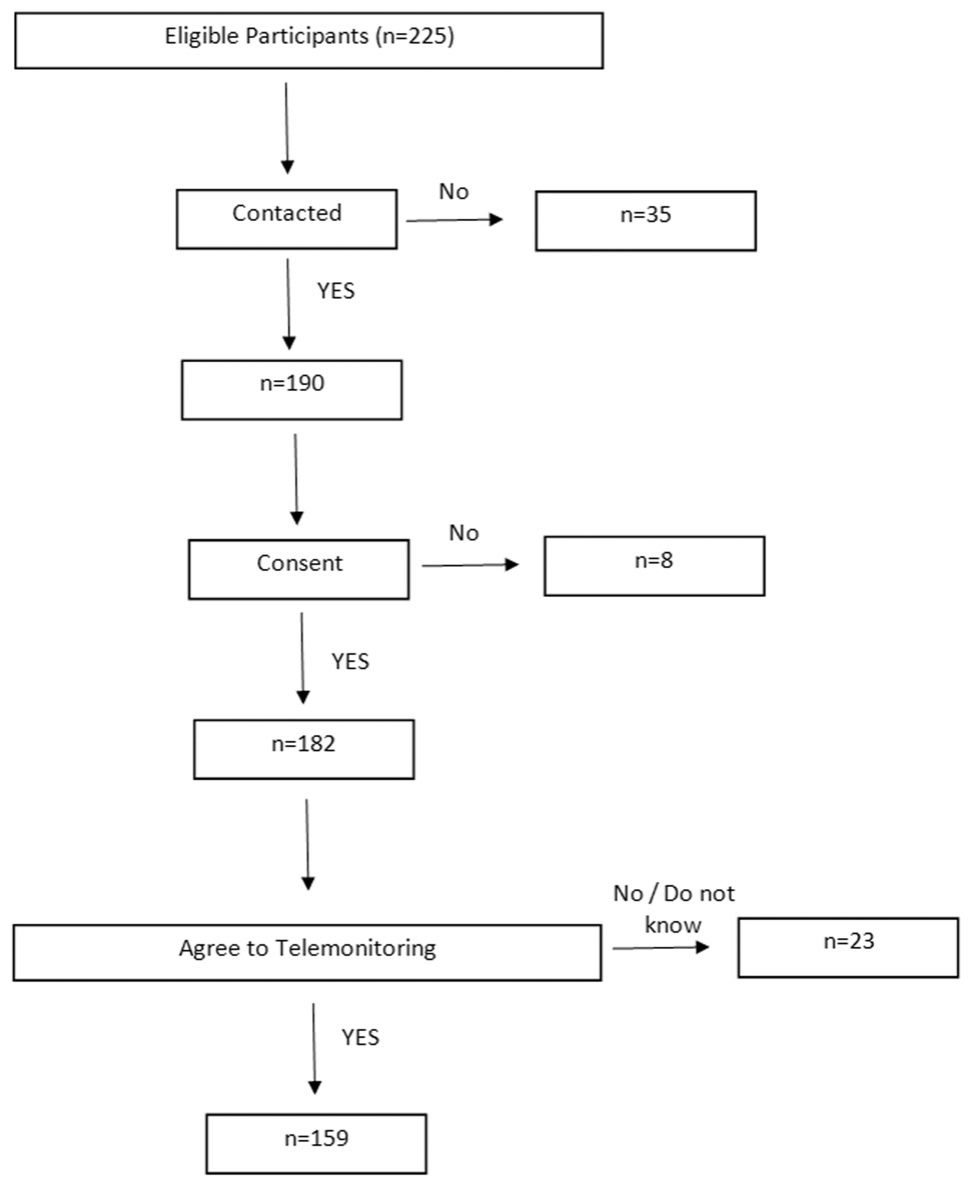
Flowchart of the steps followed in the present study.

A total of 225 patients had their appointments postponed due to the COVID-19 pandemic. We were able to contact 190, and, of these, 182 agreed to participate ( Fig. 1 ). In total, 35 patients (15.5%) could not be reached by telephone.

The mean age of the participants was 59 years, ranging from 20 to 87 years, and a median of 60 years. A total of 81.3% of the patients answered that they had internet access. Most participants accessed it mainly through their mobile phones and from their own residence. Regarding the social media platforms, they were used by 97.3% of the patients who had internet access. A total of 76.4% of the sample had the 3 most frequent urogynecologic diagnoses: mixed urinary incontinence, stress urinary incontinence, and overactive bladder. Less frequent diagnoses included pelvic organ prolapse, recurrent urinary tract infection, urinary retention, bladder pain syndrome, vesicovaginal fistula, and vesical endometriosis. A total of 159 (87.3%) participants accepted telemedicine as a form of care for their urogynecological condition; 20 (11%) patients did not accept telemedicine, preferring to be examined in person at the outpatient clinic, and 3 (1.7%) did not know how to answer this question. The acceptance of telemedicine was strongly associated with high levels of schooling, internet access, daily use of the internet, mobile data availability, and internet access from the participant's residence ( *p* < 0.001) ( Table 1 ).

**Table 1:** Sociodemographic, diagnosis, and internet accessibility according to acceptance of telemedicine care

Variables	Group 1 ( *n* = 159)	Group 2 ( *n* = 20)	*p-* value*
Age			
< 50	49	2	0.052
>51	110	18	
Education Level			
Low education	55	14	<0.01
High education	102	6	
Local of Residency			0.717
Same city of the hospital	102	14	
Metropolitan area	38	5	
Distant city	17	1	
Diagnosis/Follow-up			0.550
Urinary Incontinence ^**α**^	122	16	
Pre-operative care	3	0	
Post-operative follow-up	33	1	
Overactive Bladder	18	3	
Stress incontinence	22	1	
Mixed Incontinence	46	11	
Pelvic Organ Prolapse ^**α**^	14	3	
Pre-operative care	2	1	
Post-operative follow-up	5	0	
Conservative treatment	7	2	
Urinary retention	3	0	
Recurrent urinary tract infection	5	0	
Other	10	0	
Internet Access			<0.01
Yes	141	6	
No	17	13	
Daily Use of Internet			<0.01
Yes	123	4	
No	18	5	
Device			<0.01
Personal mobile phone	127	4	
Family's or friend's mobile phone	18	2	
Tablet	9	0	
Computer	40	2	
Place of Access			<0.01
Own residence	118	4	
Family's or friend's residence	6	1	
Workplace	9	0	
Any place with internet	45	2	

Group 1: Accepted Telemedicine; Group 2–No Accepted Telemedicine; *Chi-square and Fisher Test; ^α^ Grouped variables

A multivariate analysis was performed for the level of schooling to compare it with other demographic variables ( Table 2 ). A multivariate analysis was performed for the level of schooling to compare it with other demographic variables. Only high level of schooling was associated with the acceptance of telemedicine in both univariate (odds ratio [OR]: 5.18; 95%; CI¼1.77–15.16; *p* <0.01) and multivariate analysis (OR: 4.82; 95 CI¼1.59–14.65; *p* <0.01).

**Table 2:** Univariate and Multivariate Analysis for age, educational level, residence and diagnosis associated with telemedicine

Variables	Crude OR (95%CI)	p-value	Adjusted OR (95%CI)	p-value
Age				
> 51	0.26 (0.05-1.20)	0.086	0.4 (0.08-1.96)	0.264
Education Level				
High education	5.18 (1.77-15.16)	<0.01	4.82 (1.59-14.65)	<0.01
Local of Residency				
Metropolitan area	0.95 (0.31-2.85)	0.931	1.04 (0.32-3.34)	0.942
Distant city	2.05 (0.25-16.83)	0.500	2.36 (0.26-20.89)	0.438
Diagnosis/Follow-up				
Pelvic Organ Prolapse	0.57 (0.03-10.06)	0.702	0.63 (0.15-2.70)	0.542

OR: Odds Ratio; CI: Confidence Interval; Adjusted for: age, educational level, local of residence and diagnosis/follow-up; n = 159

## Discussion

Although telemedicine in Brazil is recent and probably underutilized in public hospitals, we found that most participants were interested and agreed to have their urogynecological condition monitored through this type of care. Among the associated factors, the level of schooling presented the strongest association with the acceptance of telemedicine. The factors involved in the non-acceptance of telemedicine included low level of schooling, lack of internet access, and lack of daily internet use. We believe our sample was representative of the studied population, once it comprised the number of patients usually seen in personal appointments for three months.

The World Health Organization (WHO) considers that telehealth plays an essential role in achieving universal access to health in a cost-effective manner, especially in the developing world. 15 Although it is believed that low- and middle-income countries can benefit more from remote access to health care services, its use for urogynecological patients has mainly been studied among educated women living in economically-developed countries. 16 The difficulties in effectively implementing its use were studied by Scott Kruse et al. 14 (2018) in a systematic review, and they included concerns about data privacy and confidentiality; the patient's age and level of schooling; computer access; bandwidth of dwelling; and resistance to change. A review 17 on the use of telehealth in urology showed similar results and mentioned access to the internet, familiarity, and ease of use with technology as barriers to its implementation on the part of the patients. The results of the present study are similar to those of other studies in the published literature. We found that the level of schooling was the most substantial limiting factor associated with the acceptance of telemedicine in our population, even though the confidence intervals in the multivariate analysis are wide, which can reduce the precision of our effect estimate. We believe this is due to the fact that lower levels of schooling are associated with lower ability to communicate through and use the technological resources available. Most of the participants in the present study were considered to have high levels of schooling, although in Brazil, 6.6% of the population (14 million people) is deemed illiterate, and most of them are users of the public health system. 18 We have observed that the schooling profile of these users has been changing in recent years and could explain the findings of the present study.

Most patients interviewed had access to the internet through a mobile phone. This corroborates with the data from 2016 by the WHO, which acknowledges that mobile phone subscription rates in low- and middle-income countries are similar to those in high-income countries, alongside much lower access to other technological sources, such as computers or fixed broadband. 14 The use of mobile technology to deliver health care has been proven to be beneficial. 19 Karageorgos et al. 20 (2019) evaluated the use of mobile technologies in developing countries, and concluded that telehealth effectively motivated patients suffering from chronic diseases to adhere to treatment, attend appointments, and improve their lifestyle habits. In urogynecology, the use of mobile technology was studied in the follow-up of conservative treatment of urinary incontinence and demonstrated positive results, with improvement in symptoms and quality of life scores. 19 20

The use of social media platforms is also becoming more popular. In the medical field, they represent a source of information to the patients on their condition and have the potential of improving the patients' satisfaction, sense of belonging, autonomy, and empowerment. 21 Alas et al. 22 (2013) analyzed the usefulness of social media in urogynecology, and concluded that a significant proportion of the information available regarding incontinence, pelvic floor prolapse, and urogynecology in the most popular platforms are not medically relevant. Therefore, physicians must incorporate this growing source of information into their daily practice and make an effort to ensure that accurate information is available to their patients. A multicenter survey conducted by Mazloomdoost et al. (2016) 21 demonstrated that 74.1% of women presenting to urogynecology practices reported having at least one social networking account, and 76.4% of the women most often used the internet for personal reasons, including medical inquiries. Almost the totality of patients with internet access in the present study accessed social media platforms regularly, and these platforms could also be used to to access and deliver healthcare.

The limitations of the present study include the fact that the interviews were performed during a period when the patients had their in-office visits canceled and no perspective for the return of the activities, which could have biased them to agree with telemedicine. Also, the invitation made by telephone call limited the recruitment of the patients, once we could not confirm the contact data previously. We did not include the non-responders in the sociodemographic analyses, which could be considered a response bias, since only patients who had access to a telephone were interviewed. We only included patients from a single center in the Southeastern region of Brazil, and we cannot extend our results to different parts of the country. We did not evaluate the efficiency and feasibility of telemedicine itself, thus, and further studies are necessary to determine its use among this population. The present study enabled us to recognize the profile of patients who could benefit from telemedicine in a public hospital in Brazil. Our next step will be to implement telemedicine care for these patients.

## Conclusion

In conclusion, the present study demonstrated that patients of a public hospital in a developing country have the resources and the desire to take part in telemedicine consultations. Internet access, daily use, access through their personal mobile phone, access from the participant's residence, and level of schooling were the factors associated with the acceptance of telemedicine in urogynecology.
